# A neutrophil elastase inhibitor, sivelestat, attenuates sepsis-induced acute kidney injury by inhibiting oxidative stress

**DOI:** 10.1016/j.heliyon.2024.e29366

**Published:** 2024-04-10

**Authors:** Wei Zhu, Yingwei Ou, Chunnian Wang, Rongcheng An, Junmei Lai, Ye Shen, Xiangming Ye, Haochu Wang

**Affiliations:** a Rehabilitation Medicine Center, Rehabilitation & Sports Medicine Research Institute of Zhejiang Province, Department of Intensive rehabilitation unit, Zhejiang Provincial People's Hospital (Affiliated People's Hospital, Hangzhou Medical College), Hangzhou, Zhejiang, 310014, China; b Emergency and Critical Care Center, Department of Emergency Medicine, Zhejiang Provincial People's Hospital (Affiliated People's Hospital, Hangzhou Medical College), Hangzhou, Zhejiang, 310014, China; c Ningbo Clinical Pathology Diagnosis Center, Ningbo 315000, Zhejiang, China; d Rehabilitation Medicine Center, Department of Radiology, Zhejiang Provincial People's Hospital (Affiliated People's Hospital, Hangzhou Medical College), Hangzhou, Zhejiang, 310014, China

**Keywords:** Sepsis, Acute kidney injury, Oxidative stress, Neutrophil elastase inhibitor, Sivelestat

## Abstract

**Background:**

Sivelestat, a selective inhibitor of neutrophil elastase (NE), can mitigate sepsis-related acute lung injury. However, the role of sivelestat in inhibiting oxidative stress and attenuating sepsis-related acute kidney injury (AKI) remains unclear. Here, we reported the effects of sivelestat against oxidative stress-induced AKI by suppressing the production of oxidative stress indicators.

**Materials and methods:**

A male Sprague-Dawley rat model of sepsis was established by cecal ligation and puncture (CLP). Sivelestat or normal saline was administered into jugular vein with a sustained-release drug delivery system. Indicators of inflammation and AKI, including white blood cells (WBC), neutrophils, lymphocytes, C-reactive proteins (CRP), procalcitonin (PCT), blood urea nitrogen (BUN), creatinine (Cr) and uric acid (UA), were assessed at 24 h post-sivelestat treatment. Indicators of liver injury, including direct bilirubin (DBIL), indirect bilirubin (IBIL), aspartate aminotransferase (AST) and alanine aminotransferase (ALT), were also assessed at 24 h post-sivelestat treatment. Indicators of oxidative stress, including superoxide dismutase (SOD), malondialdehyde (MDA) and glutathione peroxidase (GSH-Px), were assessed at 12 h and 24 h post-sivelestat treatment. At 24 h post-sivelestat treatment, H&E staining of kidney and liver tissue was performed to observe pathological alterations.

**Results:**

At 24 h post normal saline or sivelestat (0.2 g/kg body weight) treatment, WBC, neutrophil, CRP, PCT, MDA, BUN, Cr, UA, AST, ALT, DBIL and IBIL were increased, while SOD and GSH-Px were decreased, in septic rats treated with normal saline compared with that in non-septic rats treated with normal saline (all p < 0.05). The changes of these indicators were reversed in septic rats treated with sivelestat compared with that in septic rats treated with normal saline (all p < 0.05). Similar results were found regarding the levels of oxidative stress indicators at 12 h post-sivelestat treatment. The degenerative histopathological changes in both kidney and liver tissues were ameliorated upon sivelestat treatment.

**Conclusions:**

Sivelestat plays a protective role in sepsis-related AKI by inhibiting oxidative stress. Our study reveals a possible therapeutic potential of sivelestat for oxidative stress-induced AKI.

## 1 Introduction

Sepsis is a severe systemic inflammatory disease that is a lethal host response to microbial infection, and its high mortality rate is due to an imbalance of inflammatory mediators [1]. From 2004 to 2009, the incidence of severe sepsis increased by an average of 13 % per year [2]. The overall mortality rate associated with sepsis is close to 18–25 %, and the standardized mortality rate for septic patients continues to be significantly higher than the overall standardized mortality rate for intensive care units (ICUs) [3,4].

The most common acute illness-related risk factors associated with acute kidney injury (AKI) include age, cardiovascular failure, liver failure, and sepsis [5]. In particular, sepsis frequently results in multiple organ failure, including renal failure related to AKI, accounting for approximately 50 % of cases of renal insufficiency in ICU [6]. AKI during sepsis is an important and independent prognostic factor for prolonged hospitalization and in-hospital death [7], leading to increased morbidity and mortality [8]. The pathophysiological mechanisms underlying AKI due to sepsis are complex and incompletely understood, but may include inflammation, oxidative stress, microcirculatory dysfunction, and changes in the renal tubular epithelial cell response to injury [[9], [10], [11]].

Sivelestat is a selective inhibitor of neutrophil elastase (NE), which has been proved to reduce lung injury [12,13], systemic inflammatory response syndrome [14], and acute respiratory distress syndrome [14]. Other evidence shows that sivelestat can improve the survival rate of septic animals and reduce sepsis-related lung injury [15,16]. By a mouse models of ischemic AKI, a significant increase in NE expression in the renal cortex/proximal tubules was observed [17]. Li et al. [18] further reported that sivelestat could promote the survival rate of septic rats by attenuating sepsis-related renal injury and restoring the decreased mean arterial pressure (MAP) and glomerular filtration rate (GFR), as well as by reducing abnormally elevated levels of blood urea nitrogen (BUN) and neutrophil gelatinase-associated lipid transfer protein (NGAL). Sivelestat has been reported to inhibit neutrophil aggregation and reduce pro-inflammatory mediators [18]. However, to our knowledge, no study determines the roles of sivelestat in mediating oxidative stress in sepsis-related AKI. In the present study, we assessed the effects of sivelestat on sepsis-induced AKI in a rat model of sepsis induced by cecal ligation and puncture (CLP), and explored the association of oxidative stress and sepsis-related AKI after sivelestat treatment.

## 2 Materials and methods

### 2.1 Animals

Male Sprague-Dawley (SD) rats (200–250 g) were purchased from Shanghai Slack Co., Ltd. (Shanghai, China) and bred under specific pathogen-free conditions at the constant temperature of 20–25 °C and humidity of 35–70 % with a 12-h light/dark cycle, and allowed free access to food and water. All animal experiments were conducted in the laboratory animal centre located in Hangzhou Hibio, with the license number SYXK 2020-0013 (Zhejiang, China). The quality of the drinking water for the SD rats complied with the regulation of the Sanitary Standards for Drinking Water (GB5749-2006).

### 2.2 Sepsis model

**Supplementary Fig. 1:**
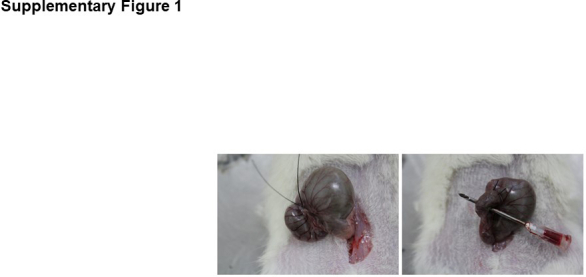
Critical steps in the CLP procedure in rats. Sepsis comprised ligation of 75 % of the cecum. Cecal puncture (‘through-and-through’) from mesenteric toward antimesenteric direction after large ligation.

SD rats were randomly divided into 3 groups (n = 20/group), including the non-sepsis group (sham-operated group), the sepsis untreated group (CLP) and the sepsis with sivelestat treatment group (0.2 g/kg body weight sivelestat sodium [Shanghai Huilun Jiangsu Pharmaceutical Co., Ltd., Shanghai, China]). The CLP procedure was performed to induce sepsis in rats according to the protocol described in previous studies [18,19]. Briefly, 0.4 % sodium pentobarbital solution (40 mg/kg; Sinopharm, Beijing, China) was administered intraperitoneally (i.p.) to anesthetize rats, and a ventral midline incision (1.5 cm in length) was made on the rats. The cecum was exposed, ligated, punctured three times with an 18-gauge needle, and then placed back into the abdomen (Supplementary Fig. 1). The rats that received a sham operation (cecum exposed through the ventral midline incision, but not ligated or punctured) were used as controls. After that, the abdominal incision was stitched with 3-0 surgical sutures, allowing rats to wake up from anesthesia.

**Supplementary Fig. 2:**
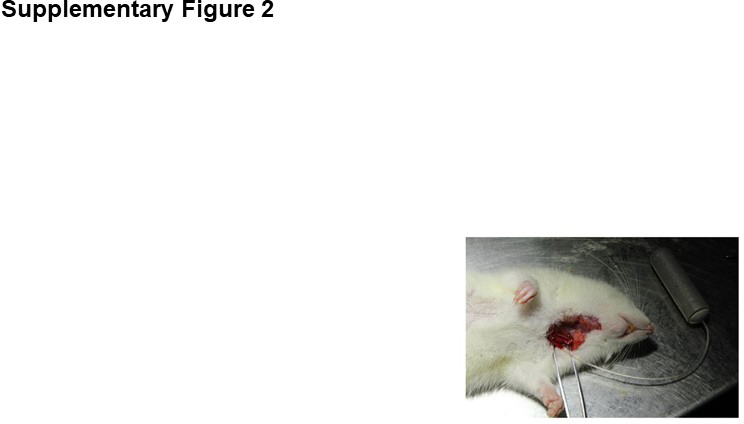
A slow-release drug pump. The jugular vein was isolated in the rats, and a slow-release drug pump (4 mg/mL) with a catheter was punctured and implanted into the jugular vein.

After surgery, the jugular vein was isolated in the sivelestat-treated group, and a sustained-release drug delivery system (4 mg/mL) with a catheter was punctured and implanted into the jugular vein (Supplementary Fig. 2). The non-septic and the septic without sivelestat treatment groups received an infusion of normal saline (vehicle) into the intraperitoneal cavity. At 24 h post-operation, all rats were sacrificed by CO_2_ euthanasia. The blood, liver, and kidney samples were collected.

### 2.3 Hematology

Whole blood was treated with anticoagulant (EDTA), and hematology parameters measured using an automatic blood cell analyzer (MEK-6318k; Nihon-kohden, Tokyo, Japan) blood cell counter, including white blood cell (WBC), neutrophil and lymphocyte.

### 2.4 Measurement of serum biochemical parameters

Blood samples were obtained from each rat at 12 and 24 h post-operation, respectively. Blood samples were centrifuged at 1000 rpm for 20 min, and then subjected to the detection of blood urea nitrogen (BUN), creatinine (Cr) levels, uric acid (UA), C-reactive protein (CRP), procalcitonin (PCT), direct bilirubin (DBIL), indirect bilirubin (IBIL), serum total bilirubin (STB), aspartate aminotransferase (AST), and serum alanine transaminase (ALT) levels using a Hitachi 7600 Autoanalyzer (Hitachi, Ltd., Tokyo, Japan) according to the manufacturer's instructions.

### 2.5 Enzyme-linked immunosorbent assay (ELISA)

ELISA was performed using commercial kits containing the commercialized buffer system according the manufacturer's instructions (Nanjing Jiancheng Bioengineering Institute, Nanjing, China). After collecting the blood samples, they were promptly centrifuged, and the supernatant (serum) was collected and stored in a −80 °C refrigerator for subsequent ELISA experiments. Total superoxide dismutase (T-SOD) activity was determined using the T-SOD assay kit (Cat. No. A001-3-2), which relies on the xanthine/xanthine oxidase (WST-1) method, measuring the production of O_2_^−^ anions. The reaction's red substance was detected with absorbance at 450 nm after incubating at 37 °C for 20 min. The amount of malondialdehyde (MDA) was determined using the MDA assay kit (Cat. No. A003-1-2), employing the thiobarbituric acid (TBA) method, with spectrophotometric measurement of absorbance at 532 nm of the red compound produced in the reaction of TBA with MDA. Glutathione peroxidase (GSH-Px) activity was determined using the GSH-Px assay kit (Cat. No. A005-1-2), which relies on the oxidative reaction between hydrogen peroxide (H_2_O_2_) and GSH, catalyzed by GSH-Px to produce oxidized glutathione and H_2_O. Additionally, GSH reacts with 5,5′-dithiobis (2-nitrobenzoic acid) (DTNB) to form a stable yellow compound, and the decrease in GSH levels is measured at 412 nm. Each assay was read in a SpectraMax®Plus 384 plate reader.

### 2.6 Histopathology

Liver and kidney specimens were fixed in 10 % formalin solution then dehydrated in ascending grades of alcohol (70 %, 95 %, and 99 % 2 min each), cleared in xylene, and embedded in paraffin. Five-micrometer-thick sections were prepared from different animal groups and stained with hematoxylin and eosin (H&E). These were then examined under a light microscope, and microphotographs were taken with a microscope attachment camera. The cellular morphology and tissue architecture changes, including focal degeneration, edema and vascular changes, as well as inflammatory cell infiltration were assessed in the kidney (renal tubules, Bowman's capsule and interstitium) and liver (hepatocytes, portal vein and sinusoid).

### 2.7 Statistical analysis

Results were expressed as the mean ± S.E.M. For a comparison of more than two groups, a one-way Kruskal-Wallis analysis of variance, followed by the Student-Newman-Keuls test for multiple comparison, was applied. Comparisons between two groups were assessed by Mann-Whitney *U* test. Spearman's correlation analysis was performed to evaluate the strength of relations between AKI-related indicators and indicators of oxidative stress. All data analysis was conducted using GraphPad Prism 8.0 software (GraphPad Software, La Jolla, CA), and SPSS version 25.0 software (IBM Corp., Chicago, IL). Values of p < 0.05 indicates statistically significant.

The sample size of rats was determined complying with the 3R principle (replacement, reduction, refinement) of animal experiments, and was calculated according to the formula: n=(Zα+Zβ)^2^ × 2σ^2^/δ^2^ [20], where Zα = 1.96^2^, Zβ = 1.28^2^, σ = 1.36 and the loss of rats per group (δ) was estimated to be 2 or 3. The calculated sample size was 12–28 rats per group. Considering our budget, we chose 20 rats for each group.

## 3 Results

### 3.1 Sivelestat decreases acute inflammation in rats with sepsis

**Fig. 1:**
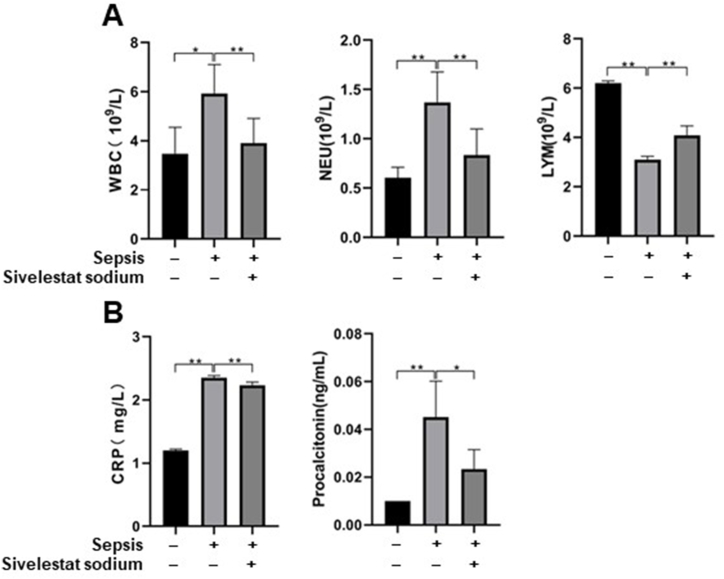
**Levels of inflammation-related indicators.** (A) White blood cells **(**WBC), neutrophils (NEU), lymphocytes (LYM) and (B) C-reactive protein (CRP) and procalcitonin (PCT) were measured at 24 h post-treatment by an automatic biochemical analyzer. The levels of these inflammatory indicators increased in septic rats receiving normal saline when compared with non-septic rats receiving normal saline. While increases of these inflammatory indicators were inhibited in the septic rats receiving sivelestat when compared with septic rats receiving normal saline. All samples were measured in duplicate. *p < 0.05; **p < 0.01.

At 24 h post-surgery, septic rats treated with normal saline had increased levels of WBC, neutrophil, CRP, and PCT compared with that in non-septic rats treated with normal saline (all p < 0.05; Fig. 1 (A, B), Supplementary Table 1). Conversely, compared to septic rats treated with normal saline, the levels of these acute inflammatory indicators significantly decreased in septic rats treated with sivelestat (all p < 0.05; Fig. 1 (A, B), Supplementary Table 1), indicating that sepsis-related acute inflammation were restored after sivelestat treatment.

### 3.2 Sivelestat preserves the kidney function in rats with sepsis

**Fig. 2:**
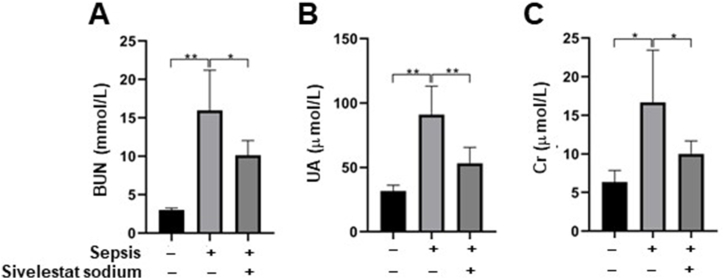
**Levels of AKI-related indicators.** BUN, Cr and UA were measured at 24h post-treatment by an automatic biochemical analyzer. The levels of these AKI-related indicators increased in septic rats receiving normal saline when compared with non-septic rats receiving normal saline. While the increases of these AKI-related indicators were inhibited in septic rats receiving sivelestat when compared with septic rats receiving normal saline. All samples were measured in duplicate. *p < 0.05; **p < 0.01.

Subsequently, we investigated whether acute inflammation-related AKI was ameliorated in septic rats at 24 h post sivelestat treatment. The results showed that AKI-related indicators, including BUN (Fig. 2A), UA (Fig. 2B) and Cr (Fig. 2C), exhibited significant increases in septic rats treated with normal saline compared with non-septic rats receiving the same treatment (all p < 0.05; Supplementary Table 2). However, the elevated AKI-related indicators were significantly reduced in septic rats treated with sivelestat compared to those treated with normal saline (all p < 0.05; Supplementary Table 2), indicating a restoration of sepsis-related AKI following sivelestat treatment.

**Supplementary Fig. 3:**
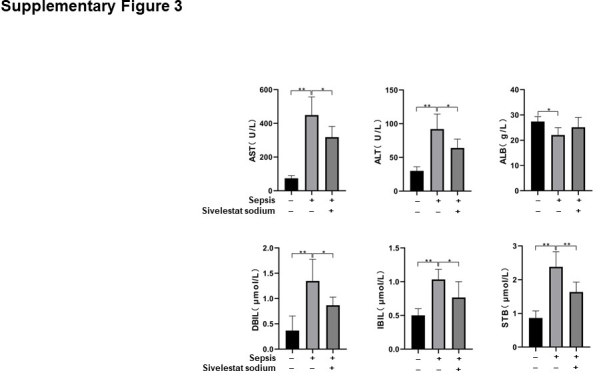
Expression of liver injury-related indicators. AST, ALT, DBIL and IBIL were measured by an automatic biochemical analyzer, and the levels of these indicators were compared between the 3 groups. *p < 0.05; **p < 0.01.

Sepsis-related liver injury was also assessed in septic rats at 24 h post-sivelestat treatment. As shown in Supplementary Fig. 3, liver injury-related indicators, including AST, ALT, DBIL, IBIL, and STB, exhibited significant increases in septic rats treated with normal saline compared with non-septic rats with the same normal saline treatment (all, p < 0.05). Conversely, septic rats treated with sivelestat demonstrated significant reduction in these liver injury-related indicators compared with septic rats treated with normal saline (all p < 0.05, Supplementary Fig. 3). These findings indicate that sivelestat treatment contributed to the restoration of sepsis-related liver injury.

### 3.3 Sivelestat inhibits oxidative stress in septic rats

**Fig. 3:**
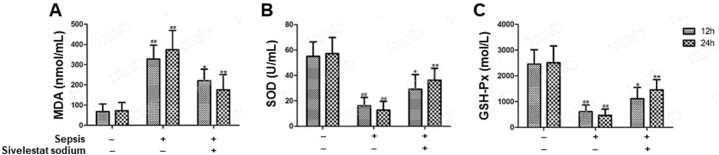
**Levels of oxidative stress-related indicators.** SOD, MDA and GSH-Px were measured at 12h and 24h post-treatment by ELISA. The levels of these oxidative stress-related indicators increased in septic rats receiving normal saline when compared with non-septic rats receiving normal saline. While the increases of these oxidative stress-related indicators were inhibited in septic rats receiving sivelestat when compared with septic rats receiving normal saline. All samples were measured in duplicate. *p < 0.05; **p < 0.01.

At 24 h post-operation, MDA (Fig. 3A) levels were significantly increased in septic rats treated with normal saline compared with non-septic rats treated with normal saline, while SOD (Fig. 3B) and GSH-Px (Fig. 3C) levels were significantly decreased in septic rats treated with normal saline compared with non-septic rats treated with normal saline (all p < 0.05). However, the level changes in these oxidative stress-related indicators were significantly restored in septic rats treated with sivelestat compared with septic rats treated with normal saline (all p < 0.05; Supplementary Table 3). Similar outcomes were also noted in these rats at 12 h post-sivelestat treatment, suggesting that sepsis-related oxidative stress was improved during the early stages following sivelestat treatment (all p < 0.05; Supplementary Table 3).

### 3.4 Oxidative stress correlates with sepsis-related AKI

**Fig. 4:**
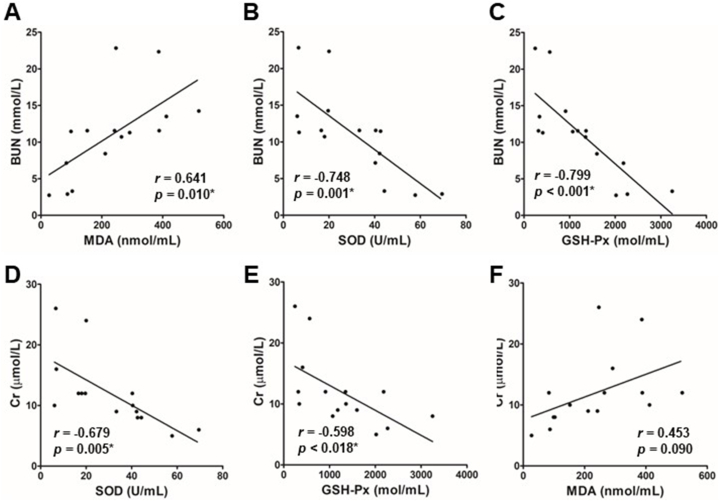
**Correlation between oxidative stress and AKI.** Spearman's correlation test was performed to analyze the data. BUN or Cr was positively correlated with MDA in septic rats, while BUN or Cr was negatively correlated with SOD or GSH-Px in these rats. *p < 0.05.

As oxidative stress was improved in septic rats treated with sivelestat, we then examined whether the indicators of sepsis-related oxidative stress were associated with indicators of AKI. As shown in Fig. 4, at 24 h post-sivelestat treatment, BUN level was positively correlated with MDA level (Fig. 4A, r_s_ = 0.641, p = 0.010) in septic rats treated with sivelestat, while it was negatively correlated with SOD (Fig. 4B, r_s_ = −0.748, p = 0.001) or GSH-Px level (Fig. 4C, r_s_ = −0.799, p < 0.001) in these rats. Similarly, Cr level was also negatively correlated with SOD (Fig. 4D, r_s_ = −0.679, p = 0.005) or GSH-Px level (Fig. 4E, r_s_ = −0.598, p = 0.018) in septic rats treated with sivelestat. However, no significant correlation between Cr and MDA was observed in septic rats treated with sivelestat (Fig. 4F, r_s_ = 0.453, p = 0.090).

### 3.5 The effect of sivelestat on tissue histopathology

**Fig. 5:**
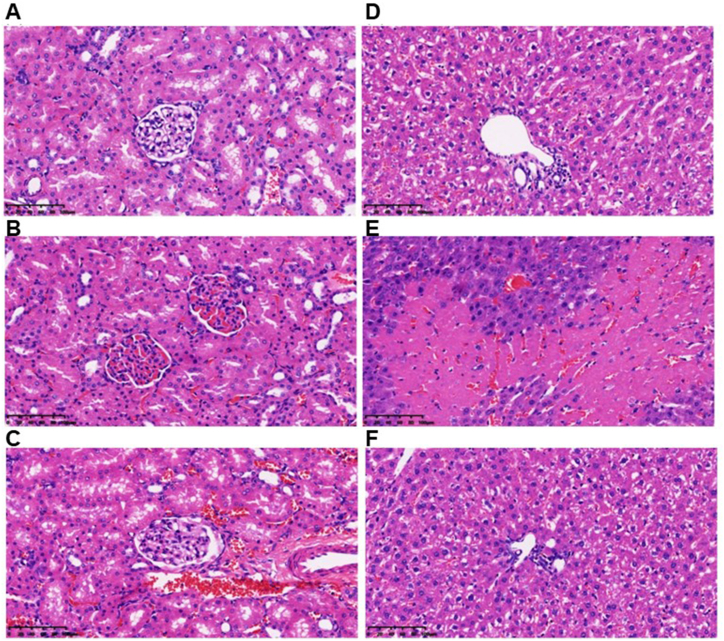
**Photomicrograph of kidney and liver tissue sections.** Hematoxylin and eosin (H&E) staining revealed the histopathological findings in the kidney (A–C) and liver (D–F). The sham-operated group showed normal morphology in the kidney (A) and liver (D). In cecal ligation and puncture (CLP) group, both the kidney (B) and liver (E) displayed tissue damage with evident inflammatory cell infiltration. In the sivelestat-treated group, there were noticeable improvements in the morphology of both the kidney (C) and liver (F). Scale bar: 100 μm.

Photomicrographs of kidney and liver tissue sections are presented in Fig. 5. In non-septic rats treated with normal saline, both the kidney (Fig. 5A) and liver (Fig. 5D) exhibited a normal morphological architecture. Conversely, the kidney of the CLP group displayed marked impairment in renal morphology, characterized by severe Bowman's capsule degeneration, tubular degeneration, and pronounced interstitial edema (Fig. 5B). However, in the septic rats treated with sivelestat group, there was a significant reduction in sepsis-induced renal damage, with mild tubular degeneration and slight interstitial edema observed (Fig. 5C).

In the liver of septic rats treated with normal saline, focal degenerative changes, edema in most areas, vascular congestion, and inflammatory cell infiltration were evident (Fig. 5E). In contrast, in the septic rats treated with sivelestat, the areas exhibiting focal degeneration were diminished, although edema and vascular congestion persisted in some regions (Fig. 5F).

## 4 Discussion

Given that numerous studies have demonstrated the protective role of sivelestat in different pathological processes, this study further explored whether sivelestat could attenuate pathophysiology and oxidative stress of sepsis-induced AKI using the CLP animal model. The main findings of this study are as follows: (i) sepsis increased the levels of markers of acute inflammation, oxidative stress, and AKI; (ii) sivelestat ameliorated sepsis-induced acute inflammation, oxidative stress, and AKI; and (iii) increased oxidative stress is correlated with sepsis-induced AKI. These findings suggest that oxidative stress contributes to the pathogenesis of sepsis-induced AKI, whereas sivelestat exerts a protective effect by inhibiting oxidative stress.

Sepsis is a systemic inflammatory response syndrome induced by infection, which commonly manifests in patients admitted to the ICU. It frequently leads to multiple organ failure, notably sepsis-related AKI, which is the primary cause of death among critically ill patients, with a mortality rate that can reach up to 70 % [21]. While the pathogenesis of sepsis-related AKI remains incompletely understood, it is primarily characterized by inflammation, oxidative stress, microcirculatory dysfunction, and alterations in the response of tubular epithelial cells to injury [9,[22], [23], [24]]. Initially, leukocytes are attracted to the vicinity of injured endothelium and promote innate immunity through cell adhesion molecules on their cell surface [25]. Subsequently, the heightened recruitment of pro-inflammatory cells continues to breach the damaged gap junctions into the surrounding tissue, perpetuating persistent inflammation [26]. In addition, inflammatory cells exhibit an increased production of inflammatory-related molecules, particularly ROS [26], which, in turn, sets off a vicious cycle of more pronounced oxidative stress, heightened inflammation, and increased vascular damage [9,25]. In line with these studies, the levels of acute inflammatory markers (WBC, neutrophil, CRP and PCT) and oxidative stress markers (MDA) were significantly increased in CLP septic rats in the present study. This demonstrated that our CLP procedure induced acute inflammation, and oxidative stress was associated with sepsis-induced AKI, with sepsis originating from polymicrobial infection [27].

Hirche et al. demonstrated that the absence of NE increased the mortality rate in mice infected with *Pseudomonas aeruginosa* [28]. In contrast, Suda et al. revealed that inhibiting NE could enhance the survival rate of rats receiving CLP, suggesting that NE abscence may offer protection against bacterial infections, although its excessive activation may have the opposite effect [15]. Moreover, sivelastat is a low molecular weight reversible NE competitive inhibitor known to improve the survival rate in septic rats by restoring arterial pressure and estimated glomerular filtration rate (eGFR) [29], as evidenced by a decrease in AKI-associated indicators BUN and Cr [18]. Several studies have reported the protective role of sivelestat in the sepsis-induced AKI pathology [18,30]. In a study conducted by Li et al. [18], sivelestat not only increased the survival rate of septic rats while preserving renal function but also inhibited macrophage infiltration, the release of pro-inflammatory mediators, and the serine/threonine kinase (Akt) signaling pathway. The present study expands upon these findings by suggesting that sivelestat can ameliorate sepsis-induced oxidative stress (SOD, MDA and GSH-Px) and AKI (BUN, Cr and UA). Of particular note, sepsis-induced oxidative stress is closely associated with the development of AKI, which may represent a promising aspect for future research into sivelestat's potential in the treatment of sepsis-induced AKI.

The evidence regarding whether renal ischemia is a primary cause of septic AKI remains a subject of controversy [31]. This ambiguity arises because certain studies have observed renal histopathological characteristics in patients who succumbed to infectious AKI that do not typically align with severe renal ischemic injury. These features include heterogeneous focal and patchy tubular damage, infrequent tubular epithelial cell death, the presence of apical vacuolization, and a slight expansion of the mesangial matrix [32,33]. Moreover, findings from sepsis models in sheep and pigs also support the notion that AKI can occur in the absence of systemic renal ischemia [34,35]. In line with histopathological investigations in human sepsis, acute tubular necrosis and tubular apoptosis do not consistently manifest as fundamental characteristics of AKI induced by sepsis [35,36]. Concerning sepsis-induced inflammation, it appears that renal microcirculation and macrocirculation may not be closely linked [31]. Notably, research in septic sheep revealed selective renal medullary tissue hypoxia occurring 12–24 h before the onset of AKI, despite increased renal cortical perfusion, blood flow, and oxygenation [37,38]. It is established that oxidative stress can stimulate the transcription of multiple genes in response to local tissue hypoxia by preventing the degradation and activation of hypoxia-inducible factors [39]. However, research on the imbalance between ROS and the host's antioxidant defense mechanisms in sepsis remains limited [9].

In the present study, we revealed that sivelestat effectively mitigated sepsis-induced oxidative stress, with increased SOD and GSH-Px, and decreased MDA, at both 12 and 24 h following sivelestat treatment. SOD and GSH-Px are enzymes known to scavenge ROS during detoxification. GSH-Px can neutralize hydrogen peroxide and lipid hydroperoxides [40]. It achieves this by utilizing reduced glutathione (GSH) as a co-substrate. SOD can catalyze the dismutation of superoxide radicals into oxygen and hydrogen peroxide [41]. It is a crucial first line of defense against superoxide-induced oxidative stress. Variations in the expression or activity of SOD and GSH-Px may affect the efficiency of ROS detoxification. In some cases, homozygous conditions may lead to more robust enzymatic activity, while heterozygous conditions could exhibit intermediate levels of activity. In addition to mitigate oxidative stress, sivelestat exhibited restorative effects on sepsis-induced AKI, as evidenced by the significant improvements in AKI markers (BUN, Cr, and UA) observed 24 h post-treatment. Furthermore, our analysis revealed evident kidney structural abnormalities, characterized by severe Bowman's capsule degeneration and interstitial edema. Remarkably, treatment with sivelestat led to a marked improvement in these kidney structural parameters. The observed effects of sivelestat in attenuating oxidative stress and restoring the pathological injury, as shown in the present study, is supported by the findings of Zhang et al. [16]. In their study, the authors showed that sivelestat effectively mitigates acute lung injury, reducing the levels of inflammatory factors, ROS and MDA, while increasing the levels of SOD and GSH-Px in lung tissues, potentially via the molecular mechanisms of JNK/NF-κB activation and Nrf2/HO-1 signaling pathway inhibition. Further investigations are warranted to reveal whether sivelestat attenuates AKI via similar molecular mechanisms.

These findings align with existing evidence indicating that sivelestat enhances the survival rates of septic rats by rectifying impairments in MAP and GFR, while also inhibiting the elevation of AKI-related markers such as BUN and Cr [18]. Ow et al. [9] proposed that sepsis-related AKI originates from the initiation of oxidative stress-induced damage to the endothelium, subsequently leading to endothelial dysfunction and the amplification of oxidative stress. Given that current treatments for septic AKI primarily offer supportive care, there is a compelling need for potential therapeutic agents that target its underlying pathophysiology. Examining sivelestat's antioxidant properties could provide valuable insights into its mechanisms of action and potential therapeutic applications beyond its known effects. It would be beneficial to explore its ability to scavenge ROS molecules or to enhance the body's endogenous antioxidant defense systems. Conducting experiments to assess its antioxidant potential through various *in vitro* and *in vivo* studies could help uncover its broader range of benefits.

Early detection of sepsis would allow for timely intervention and treatment and improve outcomes. Numerous studies have shown that increased oxidative stress occurs during the development of sepsis, resulting in decreased expression of SOD and GSH-Px and increased expression of MDA [42,43]. Since sepsis is caused by an imbalance between ROS generation and antioxidant capacity [44], the levels of oxidative markers have been widely recognized as biomarkers of sepsis over the past few decades [45,46]. The results of this study found that the MDA level in septic rats was significantly higher than that in non-septic rats at 12 and 24 h after surgery. In contrast, the levels of SOD and GSH-Px were significantly lower in septic rats than that in non-septic rats. Furthermore, these oxidative stress indicators correlated to sepsis-induced AKI at 24 h after surgery, suggesting that these indicators of oxidative stress may serve as potential biomarkers for early detection of sepsis-induced AKI.

Preclinical studies exploring inhibitors of oxidative stress in sepsis-induced AKI may hold promise for mitigating AKI [9,[47], [48], [49], [50]]. In our present study, we have demonstrated that oxidative stress likely contributes to the pathogenesis of sepsis-induced AKI. Importantly, sivelestat emerges as a key player in reducing oxidative stress, suggesting its potential clinical utility in the treatment of sepsis-related AKI. Notably, there are currently two ongoing pilot placebo-controlled RCTs in Australia investigating the effects of oxidative stress inhibitors on renal outcomes and vasopressor requirements in patients with sepsis (ACTRN12620000651987p and NCT04796636). These trials represent an exciting step forward in translating our findings into potential therapeutic strategies for patients battling sepsis-induced AKI.

There are several limitations of the present study. First, as the rat sepsis model was established using the CLP method, bacteria in rat cecum rather than the clinical bacterial strains isolated from human clinical samples were utilized. Although the clinical bacterial strains isolated from human clinical samples may be more representative for the bacterial pathogenesis in human sepsis, in rat models, bacterial strains from human clinical samples may not have been likely adapted to the rat hosts. In addition, they may not have specific virulence factors and interactions suitable for the rat immune system and gastrointestinal environment. As the rodent sepsis model has similar pathophysiological changes to human septis, we considered it could be used to establish a proof of concept before progressing to more clinically relevant models. Second, we measured serum ROS rather than the intracellular ROS to aligh with the scope of the present study. Further studies are warranted regarding the intracellular ROS measurements in the presence and absence of sivelestat to reveal the potential molecular mechanisms underlying the protective effects of sivelestat on AKI. Another notable limitation of the present study is that a single dose of sivelestat at 0.2 g/kg body weight was used. The choice of this dosage was made based on its being twice as high as the “high dose” employed by Li et al. in their 2016 study [18]. We adopted this approach due to our method of sivelestat administration to rats, which involved the utilization of a sustained-release drug delivery system (4 mg/mL) implanted into the jugular vein. This method differed from the intraperitoneal (i.p.) injection employed by Li et al. [18]. In Li's study, it was demonstrated that only the high dose of sivelestat (0.1 g/kg body weight) significantly prolonged survival, reversed the decline in mean arterial pressure, improved inulin clearance, and reduced macrophage infiltration in septic rats. Conversely, the low dose (0.05 g/kg body weight) of sivelestat exhibited similar effects on these parameters but did not achieve statistical significance. Nevertheless, despite our choice of sivelestat dosage was influenced by practical considerations, it is essential to acknowledge that varying dosages might yield different outcomes. Further investigations with a broader range of dosages could provide a more comprehensive understanding of sivelestat's effects in the context of sepsis-induced AKI.

## 5 Conclusion

Sepsis-related AKI is associated with inflammation and oxidative stress. Sivelestat, a NE inhibitor known to suppress the excessive production of inflammatory mediators, can also reduce the levels of oxidative stress-related indicators and ameliorate oxidative stress-induced AKI. The present study reveals the possible mechanisms of sivelestat against oxidative stress-induced AKI, including decreasing the level of MDA and increasing the levels of SOD and GSH-Px, and shows the possible therapeutic potential of sivelestat for sepsis-related AKI.

## Ethics approval and consent to participate

All experimental animal procedures in this study were reviewed and approved by Hangzhou Hibio Animal Care and Use Committee (IACUC NO. HB2104015).

## Consent for publication

Not applicable.

## Funding

This work was supported by grants from the Health Bureau of Zhejiang Province (2019KY314) and project of Administration of Traditional Chinese Medicine of Zhejiang province (2021ZB019).

## Data availability statement

The data used to support the findings of this study are included within the article.

## CRediT authorship contribution statement

**Wei Zhu:** Writing – original draft, Conceptualization. **Yingwei Ou:** Methodology. **Chunnian Wang:** Validation. **Rongcheng An:** Software. **Junmei Lai:** Project administration, Investigation. **Ye Shen:** Validation. **Xiangming Ye:** Writing – review & editing. **Haochu Wang:** Writing – review & editing, Supervision.

## Declaration of competing interest

The authors declare that they have no known competing financial interests or personal relationships that could have appeared to influence the work reported in this paper.
