# Supplementary material for: A neutrophil elastase inhibitor, sivelestat, attenuates sepsis-induced acute kidney injury by inhibiting oxidative stress
Source: Heliyon. 2024 Apr 10;10(8):e29366. doi: 10.1016/j.heliyon.2024.e29366 (PMC11024609; doi:10.1016/j.heliyon.2024.e29366)
Supplement: Multimedia component 1 [file mmc1.docx]

**Supplementary Table 1.** Levels of inflammation-related indicators represented in mean ± SD.

| Sepsis | − | + | + |
| --- | --- | --- | --- |
| Sivelestat sodium | − | − | + |
| WBC (10^9/L) | 3.47 ± 1.07 | 5.93 ± 1.17 | 3.91 ± 1.01 |
| NEU (10^9/L) | 0.60 ± 0.10 | 1.37 ± 0.31 | 0.83 ± 0.27 |
| LYM (10^9/L) | 6.20 ± 0.09 | 3.10 ± 0.14 | 4.09 ± 0.38 |
| CRP (mg/L) | 1.20 ± 0.02 | 2.35 ± 0.04 | 2.23 ± 0.05 |
| PCT (ng/mL) | 0.01 ± 0.00 | 0.05 ± 0.02 | 0.02 ± 0.01 |

WBC, white blood cells; NEU, neutrophils; LYM, lymphocytes; CRP, C-reactive protein; PCT, procalcitonin.

**Supplementary Table 2.** Levels of AKI-related indicators represented in mean ± SD.

| Sepsis | − | + | + |
| --- | --- | --- | --- |
| Sivelestat sodium | − | − | + |
| BUN (mmol/L) | 2.99 ± 0.28 | 16.0 ± 5.26 | 10.1 ± 1.90 |
| UA (μmol/L) | 31.7 ± 4.51 | 91.0 ± 22.2 | 53.3 ± 12.2 |
| Cr (μmol/L) | 6.33 ± 1.52 | 16.7 ± 6.77 | 10.0 ± 1.67 |

BUN, blood urea nitrogen; UA, uric acid; Cr, creatinine.

**Supplementary Table 3.** Levels of oxidative stress-related indicators represented in mean ± SD.

| Sepsis |  | − | + | + |
| --- | --- | --- | --- | --- |
| Sivelestat sodium |  | − | − | + |
| SOD (U/mL) | 12 h | 55.1 ± 11.4 | 16.0 ± 6.64 | 29.1 ± 11.6 |
|  | 24 h | 57.2 ± 12.6 | 12.7 ± 6.80 | 36.2 ± 9.47 |
| MDA (nmol/mL) | 12 h | 67.8 ± 37.8 | 327 ± 69.5 | 221 ± 56.9 |
|  | 24 h | 72.4 ± 40.1 | 374 ± 95.6 | 175 ± 75.4 |
| GSH-Px (mol/L) | 12 h | 2,463 ± 553 | 608 ± 263 | 1,113 ± 438 |
|  | 24 h | 2,510 ± 650 | 465 ± 245 | 1,456 ± 398 |

SOD, superoxide dismutase; MDA, malondialdehyde; GSH-Px, glutathione peroxidase.

**Supplementary Table 4.** Levels of liver function indicators represented in mean ± SD.

| Sepsis | − | + | + |
| --- | --- | --- | --- |
| Sivelestat sodium | − | − | + |
| AST (U/L) | 74.0 ± 16.4 | 449 ± 108 | 318 ± 64.4 |
| ALT (U/L) | 30.0 ± 6.00 | 92.0 ± 22.5 | 64.0 ± 13.2 |
| ALB (g/L) | 27.4 ± 2.00 | 22.1 ± 2.92 | 25.1 ± 3.90 |
| DBIL (μmol/L) | 0.37 ± 0.29 | 1.35 ± 0.43 | 0.87 ± 0.16 |
| IBIL (μmol/L) | 0.50 ± 0.10 | 1.03 ± 0.15 | 0.77 ± 0.23 |
| STB (μmol/L) | 0.87 ± 0.21 | 2.38 ± 0.45 | 1.63 ± 0.29 |

AST, aspartate aminotransferase; ALT, alanine aminotransferase; ALB, albumin; DBIL, direct bilirubin; IBIL, indirect bilirubin; STB, serum total bilirubin.
